# Assessment of the Oil Release and Insect Repellent Activity of Spray-Dried Gum Arabic/Citronella Oil Microcapsules

**DOI:** 10.3390/polym18020285

**Published:** 2026-01-21

**Authors:** Dilayda Kanmaz, Serkan Yildiz, Serpil Koral Koc, Gizem Manasoglu, Cansu Aras, Rumeysa Celen, Mehmet Tiritoglu, Sebnem Duzyer Gebizli, Ozgur Vatan, Esra Karaca

**Affiliations:** 1 Department of Textile Engineering, Faculty of Engineering, Bursa Uludag University, Gorukle, Bursa 16059, Türkiye; dilaydakanmaz@uludag.edu.tr (D.K.); serkanyildiz@uludag.edu.tr (S.Y.); skoral@uludag.edu.tr (S.K.K.); gmanas@uludag.edu.tr (G.M.); cansuaras@uludag.edu.tr (C.A.); rumeysa@uludag.edu.tr (R.C.); mtiritoglu@uludag.edu.tr (M.T.); sebnemduzyer@uludag.edu.tr (S.D.G.); 2 Department of Biomaterials, Graduate School of Natural and Applied Sciences, Bursa Uludag University, Gorukle, Bursa 16059, Türkiye; 3 Department of Polymer Materials, Graduate School of Natural and Applied Sciences, Bursa Uludag University, Gorukle, Bursa 16059, Türkiye; 4 Department of Biology, Faculty of Arts and Sciences, Bursa Uludag University, Gorukle, Bursa 16059, Türkiye; ovatan@uludag.edu.tr

**Keywords:** microcapsule, spray drying, citronella oil, gum arabic, release behavior, insect repellent activity

## Abstract

Essential oils are natural insect repellents, which can be microencapsulated and protected by wall materials to provide prolonged protection against insects. The protection and release of these repellents depend on various parameters, including morphology and production conditions. Herein, twenty-seven gum arabic/citronella essential oil (GA/CEO) spray-dried microcapsules were produced by using three wall-to-core ratios (3:1, 4:1, 6:1), three inlet temperatures (120, 150, 180 °C), and three feed rates (1, 2.5, 5 mL/min). The morphology, particle size, encapsulation efficiency, and release rates were evaluated. The insect repellent activity of microcapsules (0.25, 0.5, and 1 g) against *Drosophila melanogaster* flies was tested. A systematic process optimization was carried out by evaluating the effects of both emulsion concentration and process parameters on the release rates. Microcapsules with smooth surfaces and homogeneous particle sizes were produced. Encapsulation efficiency reached 90% by increasing the inlet temperature and feed rate. Slower release rates (approximately 40%) were achieved with higher concentrations of the wall material and temperatures, generally. Optimal process conditions were determined as a wall-to-core ratio of 4:1, temperatures exceeding 150 °C, and feed rates above 2.5 mL/min. The highest repellent activity achieved was 95%, indicating effectiveness of GA/CEO microcapsules as insect repellent materials.

## 1. Introduction

Essential oils and their components are odoriferous, complex natural mixtures that can be extracted from different parts of aromatic plants such as flowers, fruits, leaves, seeds, roots, barks, and stems [1,2]. In addition to their multifunctional properties, they have attracted attention for many years due to their natural and eco-friendly characteristics and have been utilized in various industries [3,4]. However, essential oils are volatile compounds that are highly sensitive to environmental factors such as temperature, light, humidity, oxygen, and interaction with other chemical components. Therefore, their industrial applications might be challenging [5,6].

Microencapsulation is an effective and preferred technology for exceeding these limits and improving the handling properties of these compounds by protecting core material, enhancing stability, and providing controlled release [7,8]. Microencapsulation is the process of creating small capsules by coating tiny solid, liquid, or gas particles with a protective wall (natural or synthetic polymers) that isolates the core compound from the external environment [9,10]. Although the application method selection depends on several factors, spray drying is one of the oldest and most widely preferred techniques, especially for encapsulating flavors and essential oils [11,12]. In addition to being a flexible, fast, continuous, repeatable, and economical process with low operating costs, the variety of encapsulating matrices, the high retention efficiency, and the stability of the compounds are also advantages of the method [13,14]. The process involves atomizing liquid droplets into a hot drying medium, leading to rapid water evaporation, which results in a quick wall formation and immediate entrapment of the core material [15,16]. Optimization of spray drying parameters is crucial, as they directly impact the encapsulation performance, properties, and quality of the final product [3,14].

GA, a natural polysaccharide, is highly favored as a wall material in microencapsulation technology due to its numerous beneficial properties. These include high solubility and low viscosity in aqueous solutions, the ability to produce stable emulsions with most oils in a wide pH range, excellent retention of volatile substances during drying, and effective film-forming capabilities [3,17]. Tupuna et al. [18] reported that GA provides higher encapsulation efficiency than maltodextrin, further highlighting its suitability for protecting sensitive bioactive compounds.

Citronella essential oil (CEO), discovered in 1910 [19], is one of the essential oils that stands out with its antimicrobial, antibacterial, antifungal, antiparasitic, antispasmodic, insect repellent, carminative, flavoring activity, and ecological pesticide properties [20,21,22]. CEO is a promising eco-friendly biopesticide due to its biodegradability, facilitating sustainable management of insect pests [23,24,25]. In addition to its insecticidal activity against various species, CEO has demonstrated strong repellent effects on *Drosophila melanogaster*, one of the most extensively used model organisms in biological research [26,27]. Instead of killing flies, CEO repels them by masking the scent that insects find attractive with its strong odor. This can be an advantage in terms of eliminating aesthetic concerns in the usage area [26]. Research has been conducted on the use of CEO, either in its pure form or as capsules, within biodegradable films, coatings, and textile structures for insect protection [28,29,30,31,32,33,34,35,36,37].

It is obtained from the leafy parts of the Cymbopogon species and has a pale to dark yellow color [38]. There are two primary sources of CEO in trade: Ceylon type, obtained from Cymbopogon nardus, and Java type from Cymbopogon winterianus. Approximately 80% of CEO consists of citronellal (33.9%), geraniol (18.1%), and citronellol (11.1%) monoterpenes [39,40]. It is utilized in various industries such as cosmetics (soap, perfumery), camping and outdoor products (sprays, candles, and lotions), food, agriculture, pharmaceuticals, textiles, medicine, veterinary, and aromatherapy due to its beneficial properties [41,42,43]. However, it has some drawbacks when used directly: it is sensitive to oxidative degradation, provides short-term protection due to its high volatility at high temperatures [44] and rapidly evaporating behavior [45], and may cause sensitivity and irritation on human skin when used directly or in high amounts [46,47]. Therefore, like many essential oils, an accurate and efficient encapsulation process is necessary to overcome its limitations [48].

In microencapsulation, the selection of wall and core materials is critical, as each wall/core combination exhibits distinct interactions requiring specific formulation and optimization of production parameters. Several studies explored encapsulating CEO using different wall materials and methods for various applications. In previous research, acacia gum [48], tamarin gum [49], gum arabic (GA)/maltodextrin, GA/whey protein concentrate powder, maltodextrin/whey protein concentrate powder [50], skimmed milk powder/whey protein concentrate [51], chitosan/gelatin [52], GA/gelatin [4,53], gelatin/maltodextrin and whey protein isolate/GA [54], gelatin/acacia gum [55], gelatin and sodium alginate [56], ethyl cellulose [57], and poly (ε-caprolactone) [58] were utilized as wall materials to encapsulate CEO. Various methods, including coacervation [4,45,46,52,53,54], electrospraying [58], emulsion extrusion [59], and spray drying [43,48,49,50,51], were used to encapsulate CEO. While these studies have contributed to the development of CEO microcapsules, most have focused on evaluating the functional properties or comparing encapsulating materials, without systematically optimizing spray drying conditions and formulation parameters.

Microcapsules find a wide range of applications in different industries, including textiles. Textile surfaces can gain different functions by application of various microcapsules. Although there are various studies in the literature on CEO microencapsulation, there is no detailed optimization of spray drying parameters and wall-to-core ratios for GA-based CEO microcapsules. Such optimization is crucial for enhancing encapsulation efficiency, morphology, and sustained release behavior for the successful application of microcapsules, particularly in functional textiles. One of the usages of these microcapsules can be insect repellency applications. However, the insect repellent activity of GA/CEO microcapsules has not been comprehensively investigated.

To address this gap, this paper presents a systematic study to determine the appropriate emulsion concentration and process parameters to obtain GA/CEO microcapsules by spray drying. To the best of our knowledge, this is the first systematic factorial investigation on both the wall-to-core ratio and spray drying parameters to optimize the production and release behavior of GA/CEO microcapsules. Unlike previous studies, in the present study, CEO was microencapsulated using GA as the sole wall material. While several studies focused only on the production of microcapsules containing CEO, this is also the first study examining the repellent efficiency of GA/CEO microcapsules against *Drosophila melanogaster*. The microcapsules were evaluated by means of their morphology, size distribution, encapsulation efficiency, and release behavior. The results were discussed by considering the effects of the production parameters. Considering these results, the sample with the optimum properties was selected. The insect repellent activity against *Drosophila melanogaster* was investigated using Y-tube tests on the selected sample. We believe that the findings of this study will provide insight for further research into the production of microcapsules via spray drying.

## 2. Materials and Methods

### 2.1. Materials

GA (Sigma-Aldrich-G9752, St. Louis, MO, USA) was utilized as the wall-forming polymer. CEO (Cymbopogon winterianus; Florame, İstanbul, Türkiye), with a density of 0.8587 g/mL, was used as the core material. Ethyl alcohol (Tekkim-TK.911015, Bursa, Türkiye), with a purity of >99.9%, and distilled water (Tekkim-TK.920047, Bursa, Türkiye) were employed as solvents. The nitrogen used in the spray drying process was supplied from a standard industrial gas source (Asalgaz, Bursa, Türkiye), with a purity of >99.9% and a moisture content of <3.0 ppm. The insect repellent activity was evaluated on the Oregon R wild-type strain of *Drosophila melanogaster*. To eliminate the effects of age and gender, 4-day-old young female flies were used. The flies were reared on culture media prepared based on the standard medium described by Lewis (1960) [60]. The medium consisted of cornmeal, sugar, dry yeast, propionic acid, orthophosphoric acid, agar, and water.

### 2.2. Preparation of the Microcapsules

The CEO was diluted in ethyl alcohol at a concentration of 15% (*w*/*v*) at room temperature. Considering previous studies [50], the amount of oil in the emulsion was kept at the maximum level. The GA, which will form the microcapsule wall, was dissolved in pure water at 70 °C by stirring on a magnetic stirrer for 5 h to be prepared at two different concentrations of 30% and 40% (*w*/*v*). Then, microencapsulation emulsion solutions were obtained by adding the CEO solution dropwise to the GA solutions cooled to 30 °C at room temperature. According to preliminary results, three different emulsion solutions with GA/CEO ratios of 3:1, 4:1, and 6:1 (*v*/*v*) were prepared using 30% and 40% GA in a 60/40 wall/core formulation and 30% GA in a 75/25 wall/core formulation, respectively. Therefore, the total solid content in 100 mL emulsion changed to 24 g, 30 g, and 26.25 g for GA/CEO ratios of 3:1, 4:1 and 6:1, respectively. The prepared emulsions were kept at rest for 6 h to observe that phase separation did not occur. Viscosity of the emulsions was measured at 100 rpm using a DV-II+ Pro Extra Brookfield Viscosimeter (Ametek Brookfield, Middleborough, MA, USA) at standard room temperature. The viscosity values for microencapsulation emulsions of 3:1, 4:1, and 6:1 were 22.4, 60.8, and 44.8 cP, respectively.

**Figure 1:**
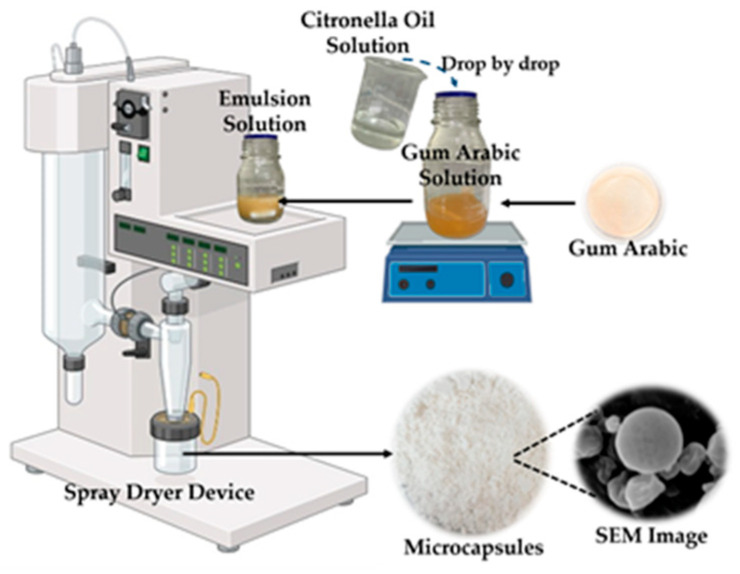
The microcapsule preparation process (the image was created with BioRender.com).

The emulsions were dried and transformed into microcapsules in an inert nitrogen environment using the Buchi S-300 Advanced Spray Dryer Device (Büchi Labortechnik AG, Flawil, Switzerland) using a 0.7/1.5 mm vertical two-fluid nozzle. The microcapsule preparation process is given in Figure 1. Three different inlet temperatures (120, 150, and 180 °C) and feed rates (1, 2.5, and 5 mL/min) were studied during production. The independent production parameters and their respective ranges were selected based on the preliminary results obtained. The spray gas and drying air flow rates were kept constant at 1800 L/h and 35 m^3^/h, respectively. The atomization air pressure was set at 5 bar, and the solution feed temperature was maintained at room temperature. Outlet temperatures were recorded below 55 °C. The microcapsule powders were collected in glass containers, and analyses were conducted immediately. Powder yield (the ratio of the weight of microcapsules produced to the weight of microcapsules that should theoretically be obtained) was determined by weighing the produced microcapsule powder with a digital precision scale. The powder yields changed in the range of 55–76%.

Variable parameters and microcapsule codes are given in Table 1.

### 2.3. Characterization of the Microcapsules

#### 2.3.1. Size Distribution Analysis

Particle size distribution was measured with a Horiba LA-960V2 Particle Size Analysis Test Device (HORIBA Ltd., Kyoto, Japan) using laser diffraction. Distilled water was used as dispersant. The refractive index of CEO was set at 1.470. Three percentiles (D10, D50, and D90), volume-weighted average size (D4,3), and span index of the volume distribution were determined. The span index was calculated according to the Equation (1).

Span index = (D_90_ − D_10_)/D_50_ (1)

#### 2.3.2. Scanning Electron Microscopy (SEM) Analysis

SEM analyses were applied to observe the morphology of the microcapsules using a Carl Zeiss AG-EVO 40XVP Scanning Electron Microscope (Carl Zeiss AG, Oberkochen, Germany). In order to make the samples conductive, the samples were coated with gold/palladium prior to the analysis.

#### 2.3.3. Fourier Transform Infrared Spectroscopy (FTIR) Analysis

GA and CEO presence in the microcapsule were examined over one sample (Sample code: 4:1_180_5) by FTIR analyses with a Shimadzu IR-Tracer100 FTIR device (Shimadzu Corporation, Kyoto, Japan). Thirty-two scans were performed in the 500–4000 cm^−1^ wavenumber range with a resolution of 4 cm^−1^.

#### 2.3.4. Thermogravimetric Analysis (TGA)

TGA thermograms of the GA/CEO microcapsules were obtained by a Shimadzu DTG-60H Thermogravimetric Analyzer (Shimadzu Corporation, Kyoto, Japan). TGA thermograms were recorded from room temperature to 500 °C at a heating rate of 10 °C/min under a nitrogen atmosphere. The thermal stability was examined on a representative microcapsule (Sample code: 4:1_180_5).

#### 2.3.5. Essential Encapsulation Efficiency

GA and CEO mounts in the microcapsules were calculated over TGA thermograms of GA/CEO microcapsules. During the experiments, samples were loaded in aluminum pans along with the standard reference aluminum. The weight loss up to 100 °C was evaluated as the water loss in the sample. The weight loss between 100 and 250 °C was measured as CEO loss. The essential encapsulation efficiency was calculated using the following Equation (2):

Essential encapsulation efficiency (%) = W_TGA_/W_CEO_ × 100 (2)

where W_TGA_ (mg) is the total CEO amount determined from TGA, and W_CEO_ is the initial amount of CEO calculated by dividing the amount of microcapsule produced to the theoretical CEO ratio in the microcapsule (this can be taken as 4 for the 3:1 wall/core, 5 for the 4:1 wall/core, and 7 for the 6:1 wall/core).

#### 2.3.6. CEO Release

During the TGA, the time-dependent release behavior of the CEO-loaded microcapsules was also investigated. For this purpose, the amount of CEO change in the microcapsules was determined over a period of 150 min at a constant temperature of 40 °C using a Shimadzu DTG-60H TGA device (Shimadzu Corporation, Kyoto, Japan) under a nitrogen atmosphere. The obtained weight changes were calculated, and a graph of the cumulative CEO release percentage over time was plotted.

#### 2.3.7. Insect Repellent Activity

*Drosophila melanogaster* is a valuable experimental model due to its key advantages, such as easy maintenance, rapid breeding, the ability to lay many eggs, and low cost. Due to its physiological, biochemical, and genetic similarities to medically important mosquitoes and flies, *Drosophila melanogaster* serves as an effective model for screening volatile insecticides against pests that pose a threat to public health (e.g., dipterans such as flies and mosquitoes) [27,61,62]. In the study, it was selected as a well-established laboratory model due to its high sensitivity to volatile compounds, ease of handling, and reproducibility in behavioral assays.

**Figure 2:**
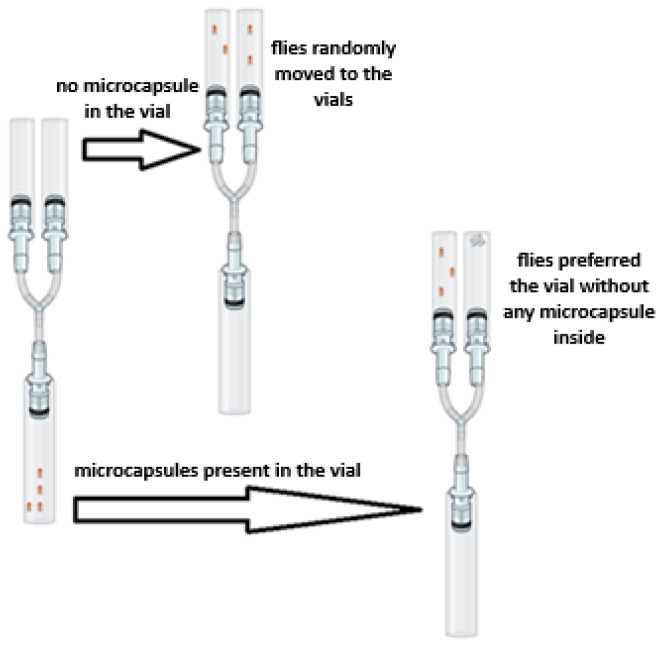
Y-tube setup used in the study (the image was created with BioRender.com).

Simonnet et al.’s [63] Y-tube test was modified to evaluate the insect repellent activity against *Drosophila melanogaster*. The female flies were placed into the experimental setup in groups of 20 individuals. The experimental apparatus is shown in Figure 2. The setup consisted of three glass vials connected to each other by a Y-shaped junction equipped with attached pipette tips.

The flies were transferred into the starting chamber, which was narrowed using sponges. The sets were prepared for three different experimental groups. The setups were kept for 24 h in a room maintained at a 12 h light/12 h dark cycle, 24 ± 1 °C temperature, and 50% humidity. Prior to the experiments, a preliminary test was carried out on two empty vials connected to the starting chamber. At the end of the period of 24 h, the average percentage of flies in the group that exited the starting chamber and moved to the right and left vials was determined. It was observed that 46.53 ± 7.50% of the flies passed to the left and 53.47 ± 7.50% of them passed to the right, confirming the random distribution. No statistical difference was found between the percentages of flies moving to the left and right in the control group (*p* = 0.421).

In the experimental groups, 0.25 g, 0.5 g, or 1 g of the microcapsules were placed in the right vial. The left vial was kept empty and used as the control. Each experimental group of 20 flies was tested separately five times. Similar to the preliminary test, the average percentages and standard deviations of flies that exited the starting chamber and moved to the vial containing the microcapsules and to the other empty vial were determined.

The statistical analyses were performed using IBM SPSS Statistics (Version 28.0). The comparisons within and between the groups were evaluated by one-way ANOVA and Tukey’s HSD test. The statistical significance level was set as *p* ≤ 0.05.

## 3. Results and Discussion

### 3.1. Morphology of the Microcapsules

**Figure 3:**
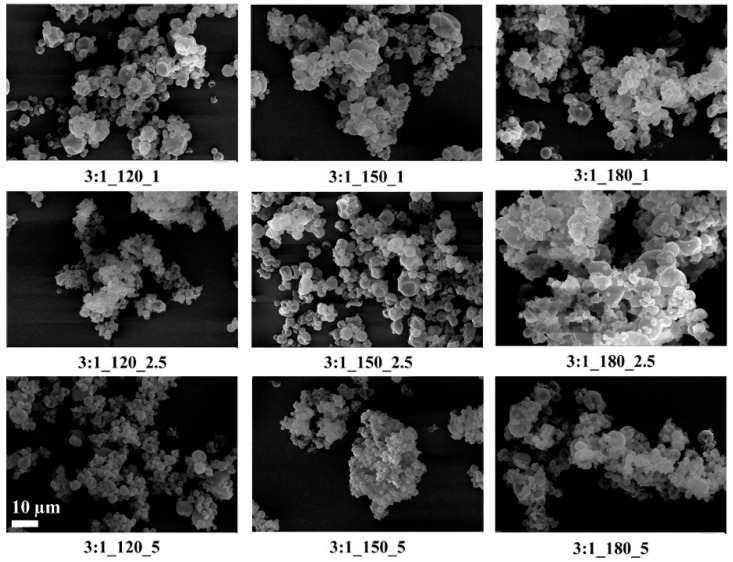
SEM images of GA/CEO microcapsules produced with 3:1 wall-to-core ratio (the scale bar shows 10 µm).

**Figure 4:**
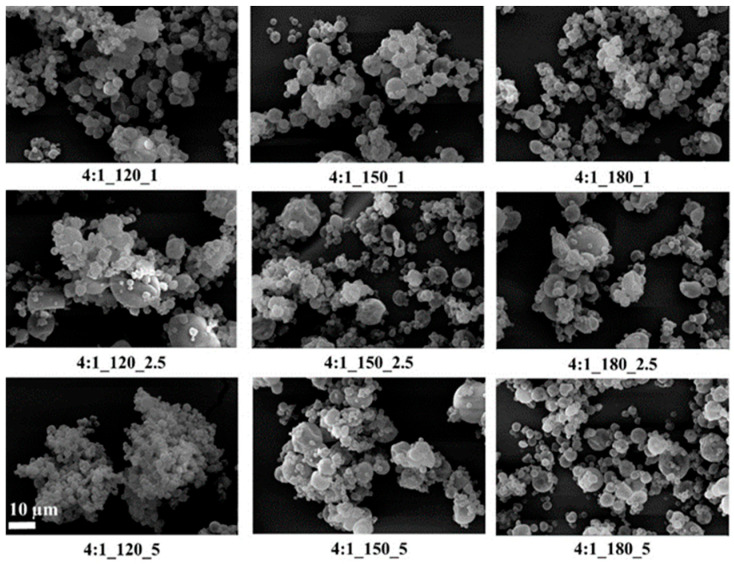
SEM images of GA/CEO microcapsules produced with 4:1 wall-to-core ratio (the scale bar shows 10 µm).

**Figure 5:**
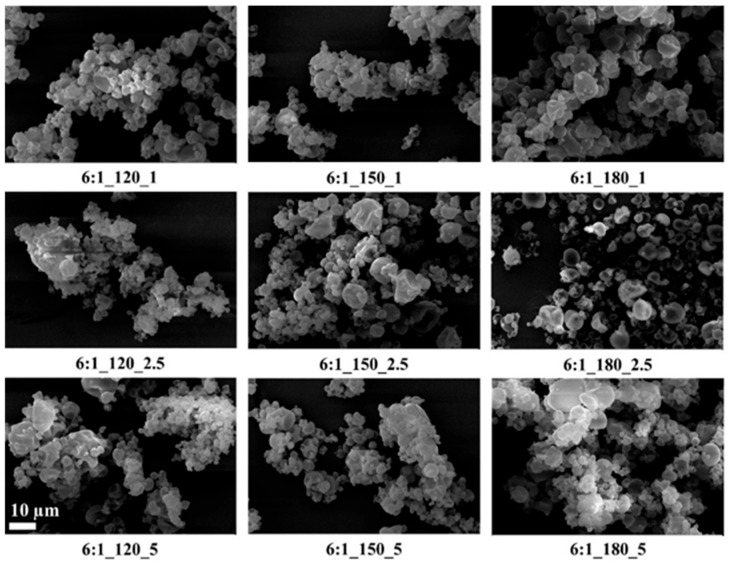
SEM images of GA/CEO microcapsules produced with 6:1 wall-to-core ratio (the scale bar shows 10 µm).

SEM images of the microcapsules are given in Figure 3, Figure 4 and Figure 5.

SEM images showed that, in general, the microcapsules aggregated, there were dents in their structures, their spherical forms were distorted, and there were variations in their size distributions. It is known that spray-dried microcapsules may have dents in their structures, and they often aggregate [9,64,65]. On the other hand, the microcapsules had smooth surfaces without any cracks or pores, which is important to increase the oil retention.

Tendency to aggregate, and defects in the spherical form on the surface depend on many parameters, such as wall thickness, emulsion viscosity, spray gas pressure/temperature/flow rate, the surface tension of the oil, and drying temperature. In particular, the presence of dents and surface irregularities in microcapsules can be attributed to viscosity, mechanical, and thermal stress properties during the spray drying process. When the external elastic deformation stress exceeds the mechanical strength of the capsule wall, stress-induced cracks or surface collapse may occur [66,67]. Rocha et al. [68] reported that the outer surfaces of microcapsules should have continuous walls without cracks, crevices, or ruptures because this feature is important to achieve lower gas permeability and better protection and retention of the core material. In the present study, microcapsules with a wall-to-core ratio of 6:1 exhibited pronounced agglomeration and irregular microcapsule surfaces, which may negatively affect their dispersibility during production and application. Although some dents and partial aggregation were observed at a 4:1 wall-to-core ratio, the capsules generally maintained spherical shapes with crack-free surfaces, suggesting more favorable morphological characteristics for practical use.

### 3.2. Chemical Structure of the Microcapsules

**Figure 6:**
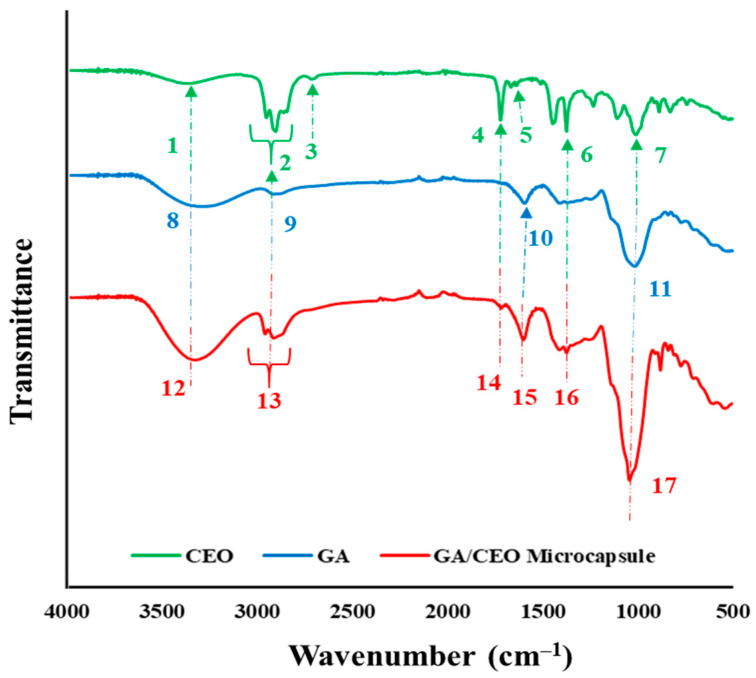
FTIR spectra of CEO, GA, and GA/CEO microcapsule (4:1_180_5).

FTIR analysis was conducted on one of the microcapsules (4:1_180_5) to identify the characteristic peaks of CEO and GA. Figure 6 and Table 2 present the infrared spectra of CEO, GA, and one of the GA/CEO microcapsules (4:1_180_5).

CEO contains various terpenes in its structure. For the spectrum of CEO, the peaks correspond to the variety of terpenes in its structure. Therefore, the peaks observed in CEO corresponds to the chemical functional groups of these terpenes, primarily citronellal, citronellol, and geraniol [46]. In the spectrum, the peak at 3375 cm^−1^ can be attributed to the O−H stretching vibration, which is related to the citronellol and geraniol in CEO. These two components are the primary alcohols of CEO and can participate in intermolecular hydrogen bonding resulting an increasing O−H bond length [69]. The peak around ~3000–2800 cm^−1^ corresponds to C−H stretching [70]. Another characteristic group of CEO is the aldehyde of citronellal. The peaks at 2725 and 1726 cm^−1^ appeared due to the H−C terminal aldehydic stretching and C=O stretching of aldhyte [69]. Other characteristic peaks of CEO at 1641, 1377, and 1008 cm^−1^ appeared due to the O−H bend, deformation of C−O−H group, and C−O stretch, respectively [55].

For GA, the characteristic bands at 3600–3000, 3000–2800, 1600, and 1000 cm^−1^ appeared due to the presence of hydrogen bonded O−H group, the presence of sugars, alkane, and aldehyde C−H stretch, stretching of C=O of the carboxylic group, and presence of glycosidic linkage, respectively [71,72].

For the GA/CEO microcapsule, the large peaks around ~3600–3300 cm^−1^, which correspond to O−H stretching vibration for CEO and hydrogen bonded O−H group for GA, is also observed in the same range. The peak which indicates the C−H stretching appeared around ~3000–2800 cm^−1^. The peaks of CEO at 1726 and 1641 cm^−1^ was seen at 1720 and 1604 cm^−1^ in the spectrum of GA/CEO, respectively. Deformation of the C−O−H group of CEO was detected at 1377 cm^−1^. Moreover, a sharpened peak at 1043 cm^−1^ was observed. This peak can be attributed to the C−O stretch of CEO, and glycosidic linkage of GA. The results indicated that the GA/CEO microcapsule presented the peaks of CEO and GA, pointing out the successful integration of CEO into GA.

### 3.3. Thermal Behavior of the Microcapsules

**Figure 7:**
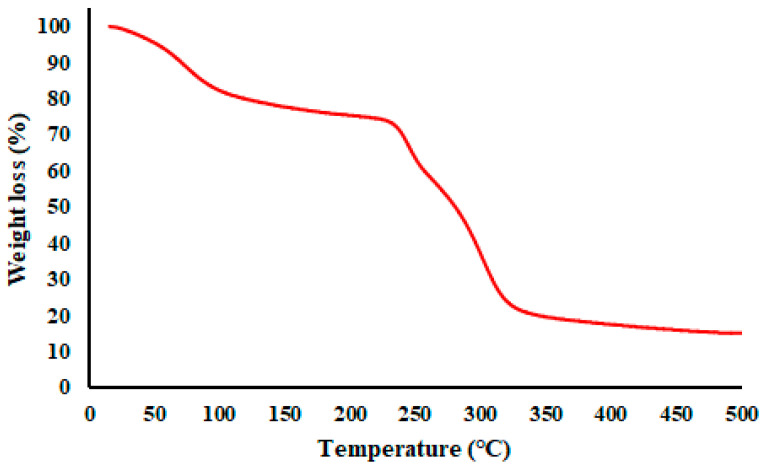
TGA thermogram of GA/CEO microcapsule (4:1_180_5).

A TGA thermogram of a representative GA/CEO microcapsule (4:1_180_5) is given in Figure 7. The TGA of the GA/CEO microcapsules revealed a multistep weight loss profile characteristic of polysaccharide-based encapsulation systems containing volatile compounds [73]. The initial weight loss observed below approximately 100 °C was attributed to the evaporation of physically adsorbed moisture associated with the hydrophilic GA wall. A second, more gradual weight loss stage occurring between approximately 100 and 230 °C was attributed to the evaporation of encapsulated CEO, indicating that CEO was retained within the microcapsule structure rather than being present solely on the surface. The major decomposition step occurring mainly between 230–350 °C corresponded to the thermal decomposition of GA. These results confirmed that the thermal behavior of GA/CEO microcapsules was mostly controlled by the polymeric wall material, enabling them to maintain structural integrity well above typical processing and storage temperatures.

### 3.4. Particle Size Analysis of the Microcapsules

The particle size analyses of the microcapsules produced under different parameters are presented in Table 3. Particle size distribution can be affected by emulsion viscosity, feed temperature, and feed rate. The span index of the microcapsules showing a unimodal distribution varied between 0.49 and 2.72. The span index, which indicates polydispersity of the microcapsules and quantifies the breadth of the particle size distribution [74,75], showed that the produced particles were homogeneous. Especially in production where the temperature was higher (180 °C), the span index value was generally around 0.49 and 0.55, which indicates low polydispersity for these capsules.

The results showed that the average sizes of microcapsules produced at wall-to-core ratios of 3:1, 4:1, and 6:1 with varying inlet temperatures and feed rates were ranged between 1 and 9 µm. Studies in the literature [76,77,78,79,80,81] have reported that the average size of microcapsules prepared via spray drying with various oils ranges from 3 to 15 µm. As the microcapsule size decreased, the increase in the tendency to agglomerate was also seen in SEM images. Upon examining Figure 2 and Figure 3, it was observed that agglomeration was more pronounced in the images corresponding to the 3:1_120_2.5 (1.02 µm) and 4:1_120_5 (1.09 µm) samples, which exhibited the lowest D50 values. This result is consistent with the information in the literature that smaller capsules tend to agglomerate more than larger ones [82]. The agglomeration of microcapsules in a matrix has a negative effect on their application potential [83]. Some studies stated that the microcapsule agglomerations acted as defects and reduced the mechanical performance [82,84].

An increase in the average size values was observed with the increase in the temperature from 120 °C to 150 and 180 °C. This result was associated with high temperatures, causing the structures to form early and not allowing them to shrink. At low temperatures, more shrunken and therefore smaller diameter particles were obtained in agreement with the literature [15,85].

In the spray drying method, emulsion viscosity is crucial in determining particle size [47]. A general increase in the average size values was observed with the increase in emulsion viscosity from 22.4 cP to 44.8 and 60.8 cP. Low viscosity emulsions such as 3:1 are easily atomized into small particles during spray drying. When viscosity increases, the fluidity of the liquid decreases, which increases the surface tension of the liquid, making it difficult for droplets to break up. This increases the average size of the microcapsules, as in 6:1 and 4:1. However, above a critical viscosity value, the interface between the core and wall may not form properly, which may reduce the size of the microcapsules [86,87].

Increasing the emulsion feed rate generally affected the average particle size. In particular, increasing the feed rate to 5 mL/min produced particles with the highest sizes. With increasing feed flow rate, the mass transfer between the formed droplets and the surrounding gas slowed down, resulting in larger particles [88,89].

The dispersibility of microcapsules is another significant factor that greatly influences their functional performance. It was indicated that better dispersibility was obtained with the larger capsules compared to smaller ones [82,90]. It was concluded that the particle size, tendency to agglomerate, and dispersibility of microcapsules influence their end-use applications, as these factors affect various properties such as controlled release, material retention, and stability [82,91,92].

### 3.5. Essential Encapsulation Efficiency of the Microcapsules

Encapsulation efficiency is defined as the ratio of the measured oil content in the microcapsules to the theoretical oil content. The encapsulation efficiency value is an indicator of the essential oil loss during the process of microencapsulation. It is a critical parameter for determining the effectiveness and quality of encapsulated oils. Higher encapsulation efficiency values are essential for more effective and sustained release. The encapsulation efficiency is significantly influenced by the characteristics of the wall/core materials, the specifications of the emulsion, and the conditions of the spray drying process [3,17]. Studies have reported efficiencies exceeding 70% for various essential oils [50], whereas CEO has been capsulated with efficiencies ranging between 65 and 70% [51].

**Figure 8:**
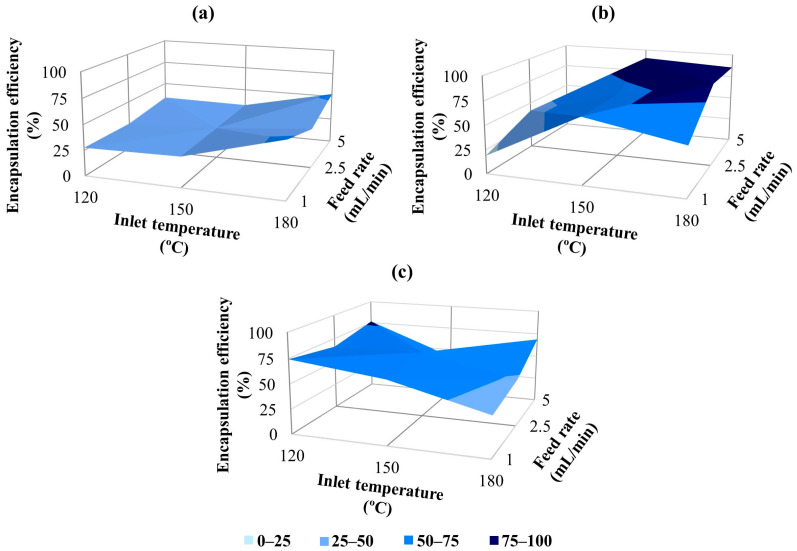
Encapsulation efficiency results of GA/CEO microcapsules with wall-to-core ratio; (**a**) 3:1, (**b**) 4:1, (**c**) 6:1.

TGA was commonly used in the literature to determine encapsulation efficiency in systems where a volatile component was used within a wall material [93,94]. Figure 8 presents the encapsulation efficiency results as a function of the wall-to-core ratio in the microcapsules. In this study, encapsulation efficiency for the microcapsules produced under different parameters ranged approximately from 20 to 90%. Too low or high temperatures can adversely affect the efficiency of oil in microcapsule production. When the air inlet temperatures are low, it becomes more challenging and delayed to form a solid membrane layer on the surface of the droplets that are sent from the atomizer to the spray drying chamber. As a result, the essential oils within the droplets evaporate more easily, leading to a decrease in efficiency [3,9]. When examining the effect of the inlet temperature on encapsulation efficiency, it was observed that efficiency generally increased with rising temperature. Encapsulation efficiency values at 180 °C were usually higher than those at 150 °C for constant wall concentration and feed rates; however, the lowest results were generally observed at an inlet temperature of 120 °C. Similarly, many researchers working on the encapsulation of various oils noted that the efficiency improved with increasing inlet temperature [95,96,97]. Higher air temperature shortens the time required for crust formation, which prevents oil from spreading further onto the particle surface. This results in maximum retention of volatiles [98,99]. The 180 °C inlet temperature, which generally provides the highest encapsulation efficiency results, also coincides with the temperature range (160–220 °C) in the literature, which is expressed as the sufficiently high inlet temperature leading to the rapid formation of the semi-permeable membrane on the droplet surface [17].

Feed rate is one of the most important parameters affecting microcapsule formation in the spray drying method. It should be adequate to ensure that the liquid evaporates before the particles contact the drying chamber wall [12]. Generally, better encapsulation efficiency results were obtained at 2.5 and 5 mL/min feed rates compared to 1 mL/min, while keeping the wall-to-core ratio and inlet temperature constant. These findings were consistent with studies reporting higher efficiency by increasing the feed rate [100,101]. Alvarenga Botrel et al. [102] also stated that the improvement in oil retention at high feed rates may be due to the rapid formation of the semi-permeable membrane due to the higher solids content in the drying chamber. However, as an exceptional case, the highest efficiency value was observed at the lowest feed rate of 1 mL/min in samples produced at 180 °C temperature with a 3:1 wall/core ratio. It is also stated in the literature [102] that high encapsulation efficiency can be achieved in high inlet temperature/low feed rate combinations.

It is crucial to determine the amount of wall material required to enhance the retention of essential oils and prevent changes caused by oxidation and chemical interactions or volatilization [12,102]. Research indicates that the key factor affecting the retention of volatiles and the encapsulation efficiency during spray drying is the concentration of dissolved solids in the feed emulsion. When comparing wall-to-core ratios of 3:1 and 6:1, both using 30% GA, the samples with a 6:1 ratio exhibited higher encapsulation efficiency results than those with 3:1. According to Frascareli et al. [103], oil concentration is a critical factor affecting efficiency; specifically, a higher oil concentration typically leads to lower efficiency. Considering that the 6:1 emulsions contained less oil and more gum than the 3:1 samples, these findings align with the existing literature. The effects of oil and gum concentrations on encapsulation efficiency and retention can also be related to emulsion viscosity to some extent. As stated in the Materials section, the viscosity of 4:1 emulsions prepared with 40% GA was higher than that of 3:1 and 6:1 emulsions due to the higher solid content (60.8 cP). The highest encapsulation efficiency results were generally achieved with the 4:1 wall-to-core ratio. This can be related to the fact that the higher viscosity emulsions can lead to decreased internal circulations and oscillations of the droplets and reduce the time needed for crust formation. Additionally, in emulsions with higher solid content, oil diffusion to the drying particle surface becomes more difficult, resulting in enhanced oil retention and efficiency [15,17].

Compatible with SEM analysis, microcapsules with smooth, spherical, and crack-free surfaces, especially those produced at moderate temperatures and with a 4:1 wall-to-core ratio, showed higher oil retention. Deformed or agglomerated particles observed at low temperatures or with low-viscosity emulsions exhibited lower efficiency. These morphological defects likely increased surface permeability, promoting oil loss during drying. Thus, particle morphology and size directly influenced the encapsulation efficiency.

### 3.6. Oil Release from the Microcapsules

The release rate of CEO from microcapsules is influenced by various factors such as the properties of GA and CEO, the structure and sizes of the microcapsules, and the interaction between GA and CEO. The release process occurs in three stages: (i) gradual gasification of CEO, (ii) burst release of CEO, and (iii) CEO diffusion and completion of release [85].

**Figure 9:**
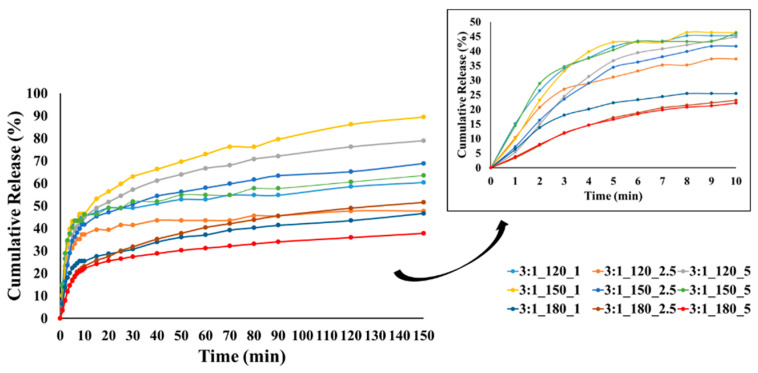
CEO release from GA/CEO microcapsules produced with 3:1 wall-to-core ratio (the inset shows the CEO release within the first 10 min).

**Figure 10:**
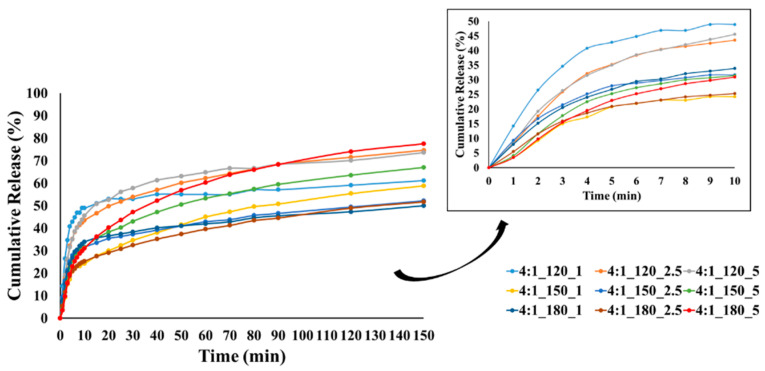
CEO release from GA/CEO microcapsules produced with 4:1 wall-to-core ratio (the inset shows the CEO release within the first 10 min).

**Figure 11:**
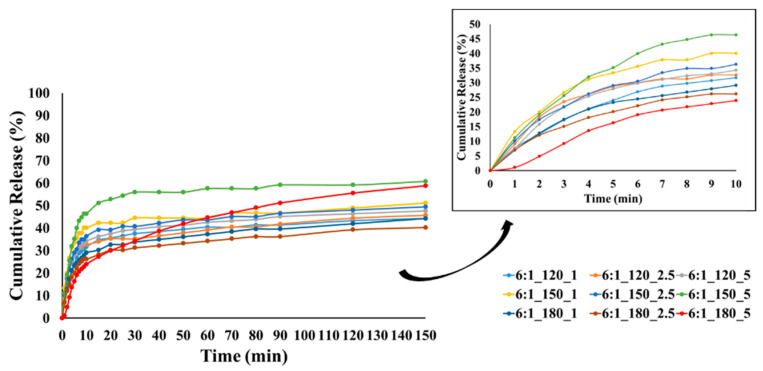
CEO release from GA/CEO microcapsules produced with 6:1 wall-to-core ratio (the inset shows the CEO release within the first 10 min).

In this study, TGA was selected as an indirect but reliable method to monitor the volatilization-driven release of CEO, which is dominated by evaporation rather than dissolution in aqueous media. TGA was employed to distinguish weight loss regions associated with moisture and essential oil evaporation and to evaluate relative trends among formulations rather than to derive absolute release kinetics. Figure 9, Figure 10 and Figure 11 show the cumulative release profiles of CEO from microcapsules prepared with wall-to-core ratios of 3:1, 4:1, and 6:1, respectively. It could be seen that the microcapsules exhibited an initial burst release ranging approximately between 22 and 48% in the first 10 min. Although cumulative burst release reached up to ~50% for all groups, 4:1 and 6:1 showed lower initial burst release rates in general. The initial burst release is affected by various parameters such as encapsulation efficiency, polymer relaxation, cross-linking between oil and wall material, oil content on or close to the surface of microcapsule, pore size and distribution of the wall material, wall thickness, incomplete removal of solvent, etc. [104,105,106].

At lower temperatures, less crosslinking, poor wall formation, or higher wall porosity may occur, allowing the essential oil to diffuse more easily to the surface and escape rapidly, resulting in higher burst release. In contrast, denser and more compact wall structures can be formed at higher temperatures, reducing the burst release. Our results showed that most of the microcapsules produced at temperatures below 180 °C exhibited higher burst release, which may be related to these factors.

The release profiles gradually reached equilibrium under the effect of diffusion with different release rates at the end of 150 min. The release rates of CEO varied between 37.8 and 89.5%, 51.7 and 77.5%, and 40.3 and 60.8% for the microcapsules with a wall-to-core ratio of 3:1, 4:1, and 6:1, respectively. It is evident that the release rate of microcapsules with a wall-to-core ratio of 3:1 remained within a relatively wide range, while 4:1 and 6:1 exhibited similar and narrower ranges. Although 6:1 was expected to have higher release rates due to its surface characteristics (Figure 5), they showed a slower release possibly due to the stronger interactions between GA and CEO [104]. Based on these results, it could be concluded that wall-to-core ratios of 4:1 and 6:1 were promising for insect repellency applications.

The duration of protection provided by CEO can vary significantly based on several factors, including mosquito species, formulation, concentration, and experimental conditions. Free CEO typically offers a deterrent effect lasting between 1 and 5 h [107,108,109]. In contrast, formulated or encapsulated systems may extend this effect to one or more days [30].

In the literature, Manaf et al. [110] prepared CEO microcapsules by a coacervation method using GA and gelatin as wall materials. They measured CEO release by Gas chromatography/mass spectrometry (GC/MS) analysis and observed an initial release of 30%, which reached 90% after 5 h. In another study, Solomon et al. [45] investigated the release behavior of Gelatin/CEO microcapsules by GC/MS analysis and achieved a burst release of 30% in 1 h and a total release of 70% at the end of 10 h. In our study, we obtained relatively high and a wide range of burst and total release rates depending on the core-to-wall ratio and production parameters. Moreover, the release behavior was investigated at 40 °C to simulate real-use conditions, which also accelerated the release rate. Murtaza et al. [59] produced CEO-loaded nanofibers and obtained 5% of release over 18 h due to the nanofiber matrix structure. Similarly, GA/CEO microcapsules can be incorporated into the nanofiber matrix for a slower release rate, and efficient insect repellency can be achieved.

### 3.7. Insect Repellent Activity of the Microcapsules

**Figure 12:**
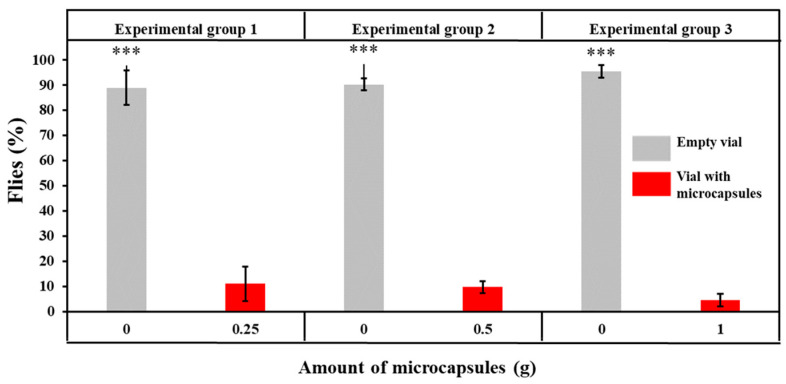
The results of insect repellent activity and statistical comparison between the vial containing microcapsules and empty vial within group (***: *p* < 0.001).

The results of insect repellent activity obtained from Y-tube tests are presented in Figure 12.

The flies repelled by GA/CEO microcapsules and moving into the empty vial were determined as 88.99 ± 6.86%, 90.23 ± 2.37%, and 95.49 ± 2.53%, whereas the flies moving into the vial containing microcapsules were counted as 11.01 ± 6.86%, 9.77 ± 2.37%, and 4.51 ± 2.53%, for 0.25 g, 0.5 g, and 1 g of microcapsules, respectively. It was determined that the percentages of flies repelled by GA/CEO microcapsules (moving to the empty vial) in each experimental group were significantly higher than the flies that did not avoid the microcapsules (*p* < 0.001). Furthermore, no statistically significant effect of microcapsule amount on the fly repellent activity was observed (*p* = 0.086). It is hypothesized that the absence of statistical significance between microcapsule quantity and avoidance behavior may be attributable to the flies demonstrating responses that approximate their maximum avoidance response. Olfactory receptor saturation in *Drosophila melanogaster* and concentration-independent odor response formation mechanisms might also be influential in this outcome [111].

Although an increase in microcapsule amount from 0.5 g to 1 g resulted in a twice higher CEO loading, no statistically significant enhancement in repellency was observed between these two formulations. Both samples exhibited repellency efficiencies exceeding 90%, a level that has been reported in the literature [112,113] as an effective threshold for volatile plant-based repellents. CEO is a volatile essential oil and repels the insects by its scent. This plateau in repellency efficacy suggested that once odor or bioactive concentrations exceed a certain perception threshold, insects did not exhibit stronger avoidance responses with further increases in the volatile compound release. From an application perspective, it was concluded that 0.5 g of microcapsules was sufficient to achieve optimal repellency, while further increments increased the material usage without a functional benefit.

*Drosophila melanogaster* possesses detector receptors capable of identifying different repellents, and these receptors play a critical role in avoidance behavior. Du et al. [114] demonstrated that citronellal was a direct agonist of the homologous TRPA1 receptor shared by *Drosophila melanogaster* and *Anopheles gambiae*, being one of the most efficient malaria vectors. Based on the results obtained with the fruit fly, *Drosophila melanogaster*, it was thought that the developed material might also have promising protection potential against higher-risk organisms such as mosquitoes, due to their genetic similarities.

## 4. Conclusions

Herein we presented a systematic study to determine the appropriate emulsion concentration and process parameters to obtain GA/CEO microcapsules by spray drying. CEO, being an interesting additive for several applications, was successfully encapsulated in GA using the spray drying method. Process parameters, such as the wall-to-core ratio, inlet temperature, and feed rate, were investigated to determine their impacts on the properties of the microcapsules. Apart from the existing literature, the study presents a systematic optimization and can be a guide for the selection of production parameters of the CEO microcapsules with suitable properties for use in many applications where insect repellency is needed.

The results indicated that high inlet air temperatures (150 and 180 °C) and high emulsion feed rates (2.5 and 5 mL/min) were the best spray drying conditions for the encapsulation of CEO in GA, considering the encapsulation efficiency. Also, the highest encapsulation efficiency values (85.5–90.5%) were obtained with a wall-to-core ratio of 4:1. Viscous emulsions and high inlet temperatures in the microencapsulation process resulted in larger particle sizes. Despite some agglomeration and dents in the capsule images, generally, spherical shapes with crack-free surfaces were achieved. The burst release values of all the samples at the end of the first 10 min were approximately 50% for all groups, and the release profiles gradually reached equilibrium under the effect of diffusion, with varying release rates at the end of 150 min.

The findings of the study indicated that the microcapsules with larger sizes presented slower release rates and higher encapsulation efficiencies. Being important for its mosquito-repellent properties, sustained and prolonged release of CEO is expected for a long-term effect. One of the limitations of the CEO microcapsules obtained in this study could be the relatively high release rates. Based on the short-term release profile analyzed in the study and the relatively high burst release results observed, it is believed that the produced GA/CEO microcapsules could be effectively utilized as an eco-friendly short-term deterrent for insects around food preparation areas, waste management applications, and drains. For applications requiring longer-acting release, it is suggested that material-integrated systems represent one of the most promising methods for CEO-based repellents. Future studies could investigate incorporating these optimized microcapsules into nanofibrous mats or coatings for potential use in outdoor textile applications.

Considering higher encapsulation efficiencies, lower initial burst release rates in a narrow range, the microcapsules produced with a 4:1 wall-to-core ratio at high temperatures can be a promising alternative for a sustained release compared to other samples. In addition, the insect repellent activity against *Drosophila melanogaster* fly was evaluated by Y-tube tests. Minimum 0.5 g of GA/CEO microcapsules provided the threshold value (≥90%), pointing out the insect repellent property.

These findings are expected to contribute to the design of optimized microcapsule systems for industrial applications and provide insights for future research on essential oil encapsulation using spray drying technology. The insect repellent activity results also indicated the potential of GA/CEO microcapsules as an insect repellent material.

## Figures and Tables

**Table 1:** Process parameters and sample codes.

Sample Code	Wall/Core Ratio (*v*/*v*)	Inlet Temperature (°C)	Feed Rate (mL/min)
3:1_120_1	3:1	120	1
3:1_120_2.5	3:1	120	2.5
3:1_120_5	3:1	120	5
3:1_150_1	3:1	150	1
3:1_150_2.5	3:1	150	2.5
3:1_150_5	3:1	150	5
3:1_180_1	3:1	180	1
3:1_180_2.5	3:1	180	2.5
3:1_180_5	3:1	180	5
4:1_120_1	4:1	120	1
4:1_120_2.5	4:1	120	2.5
4:1_120_5	4:1	120	5
4:1_150_1	4:1	150	1
4:1_150_2.5	4:1	150	2.5
4:1_150_5	4:1	150	5
4:1_180_1	4:1	180	1
4:1_180_2.5	4:1	180	2.5
4:1_180_5	4:1	180	5
6:1_120_1	6:1	120	1
6:1_120_2.5	6:1	120	2.5
6:1_120_5	6:1	120	5
6:1_150_1	6:1	150	1
6:1_150_2.5	6:1	150	2.5
6:1_150_5	6:1	150	5
6:1_180_1	6:1	180	1
6:1_180_2.5	6:1	180	2.5
6:1_180_5	6:1	180	5

**Table 2:** Functional groups of CEO and GA.

Peak Number	CEO	
	Specific Wavenumber (cm^−1^)	Functional Group—Chemical Bond
1	3600–3300	O−H stretching vibration
2	3000–2800	C−H stretching
3	2725	H−C terminal aldehydic stretching
4	1726	C=O stretching of aldehyde
5	1641	O−H bend
6	1377	deformation of C−O−H group
7	1008	C−O stretch
	**GA**	
8	3600–3300	hydrogen bonded O−H group
9	3000–2800	C−H stretching
10	1600	stretching of C=O of the carboxylic group
11	1000	glycosidic linkage
	**GA/CEO microcapsule**	
12	3600–3300	O−H stretching vibration and hydrogen bonded O−H group
13	3000–2800	C−H stretching
14	1720	C=O stretching of aldehyde
15	1604	stretching of C=O of the carboxylic group
16	1377	deformation of C−O−H group
17	1043	C−O stretch and glycosidic linkage

**Table 3:** Particle size analysis results.

Sample Code	Average Size (µm)	D10 * (µm)	D50 * (µm)	D90 * (µm)	Span Index
3:1_120_1	2.84	1.03	2.09	5.47	2.12
3:1_120_2.5	1.39	0.51	1.02	2.35	1.80
3:1_120_5	1.75	0.79	1.41	3.01	1.57
3:1_150_1	1.85	0.86	1.58	3.22	1.49
3:1_150_2.5	6.13	1.00	1.95	5.69	2.41
3:1_150_5	4.86	0.57	1.19	3.33	2.32
3:1_180_1	7.72	5.94	7.57	9.78	0.51
3:1_180_2.5	7.95	5.96	7.62	9.85	0.51
3:1_180_5	8.10	6.05	7.93	10.42	0.55
4:1_120_1	1.01	0.97	1.69	3.44	1.46
4:1_120_2.5	2.69	1.03	2.03	5.17	2.04
4:1_120_5	1.28	0.57	1.09	2.19	1.49
4:1_150_1	7.76	5.93	7.59	9.85	0.52
4:1_150_2.5	8.03	6.00	7.87	10.29	0.55
4:1_150_5	3.43	1.10	2.29	7.33	2.72
4:1_180_1	1.61	0.80	1.43	2.65	1.29
4:1_180_2.5	8.72	6.74	8.56	11.03	0.50
4:1_180_5	3.53	1.14	2.56	7.27	2.39
6:1_120_1	1.48	0.85	1.35	2.29	1.07
6:1_120_2.5	1.74	0.66	1.55	3.07	1.55
6:1_120_5	2.35	1.04	1.91	4.18	1.64
6:1_150_1	2.56	0.83	1.57	4.91	2.60
6:1_150_2.5	1.21	0.11	1.14	1.72	1.41
6:1_150_5	1.54	0.80	1.42	2.47	1.18
6:1_180_1	8.64	6.60	8.48	11.01	0.52
6:1_180_2.5	7.32	5.53	7.19	9.33	0.53
6:1_180_5	8.73	6.78	8.58	11.01	0.49

* D10, D50, and D90 values indicate the size below 10%, 50%, and 90% of the cumulative volume, respectively.

## Data Availability

The original contributions presented in the study are included in the article, further inquiries can be directed to the corresponding author.
